# Retinal pathology and skin barrier defect in mice carrying a Stargardt disease-3 mutation in elongase of very long chain fatty acids-4

**Published:** 2007-02-26

**Authors:** Anne McMahon, Igor A. Butovich, Nathan L. Mata, Martin Klein, Robert Ritter, James Richardson, David G. Birch, Albert O. Edwards, Wojciech Kedzierski

**Affiliations:** 1 Department of Ophthalmology, University of Texas Southwestern Medical Center, Dallas, TX; 2 Sirion Therapeutics, San Diego, CA; 3 Retina Foundation of the Southwest, Dallas, TX; 4 Department of Pathology and Molecular Biology, University of Texas Southwestern Medical Center, Dallas, TX; 5 McDermott Center for Human Growth and Development, The University of Texas Southwestern Medical Center, Dallas, TX; 6 Institute for Retina Research, Dallas, Texas

## Abstract

**Purpose:**

Autosomal dominant Stargardt disease-3 (STGD3) is caused by mutations in elongase of very long chain fatty acids-4 (*ELOVL4*). The goal of this study was to generate and characterize heterozygous and homozygous knockin-mice that carry a human STGD3 pathogenic mutation in the mouse *Elovl4* gene.

**Methods:**

Recombinant Stgd3-knockin mice were generated using a DNA construct which introduced a pathogenic five-base pair deletion and two point mutations in exon 6 of the *Elovl4* gene. Stgd3-mouse genotypes were confirmed by Southern blot analysis and expression of wild-type (wt) and mutated Elovl4 mRNAs assayed by nuclease protection assay. The retinal phenotype of heterozygous Stgd3 mice was characterized by morphological studies, elecroretinographic (ERG) analysis and assay of lipofuscin accumulation. Homozygous Stgd3 mice were examined for both retinal and gross morphology. They were also analyzed for skin morphology and skin barrier function, and for epidermal lipid content using high performance liquid chromatography (HPLC) combined with mass spectrometry (MS).

**Results:**

The *Stgd3* allele codes for a truncated mouse Elovl4 protein, which also contains the same aberrant 8-amino acid C-terminus encoded by the human pathogenic STGD3 allele. Heterozygous Stgd3 mice expressed equal amounts of both wt and mutant Elovl4 mRNAs in the retina, showed no significant changes in retinal morphology, but did show accumulation of lipofuscin and reduced visual function. Homozygous Stgd3 mice were born with an expected Mendelian frequency, without any initial gross anatomical or behavioral abnormalities. By 6-12 h postpartum, they became dehydrated and died. A skin permeability assay detected a defect in epidermal barrier function. Homozygous mutant epidermis expressed a normal content of mutated Elovl4 mRNA and contained all four epidermal cellular layers. HPLC/MS analysis of epidermal lipids revealed the presence of all barrier lipids with the exception of the complete absence of acylceramides, the critical lipids for barrier function of the skin.

**Conclusions:**

The generated Stgd3-knockin mice are a genetic model of human STGD3 and reproduce features of the human disease: accumulation of lipofuscin and reduced visual functions. Homozygous Stgd3 mice showed a complete absence of acylceramides from the epidermis. Their absence suggests a role for Elovl4 in acylceramide synthesis, and in particular, a role in the synthesis of the unique very long chain C30-C40 fatty acids present in skin acylceramides.

## Introduction

Three independent mutations in the gene coding elongase of very long chain fatty acids-4 (ELOVL4) have been shown to cause Stargardt disease-3 (STGD3), a juvenile-onset, autosomal dominant macular degeneration [1-4]. This hereditary disease is characterized by accumulation of lipofuscin and gradual loss of central vision [5,6]. ELOVL4 is homologous with five other enzymes known to elongate very long chain fatty acids, but its substrate/product specificity and physiological role are currently unknown. All three human STGD3 pathogenic mutations lead to a truncated ELOVL4 protein deprived of its C-terminal sequence containing a signal for retention in the endoplasmic reticulum (ER) [1].

In mammals, all fatty acids longer than 16 carbon atoms are either derived from dietary sources or are synthesized in the ER [7]. The synthesis requires four enzymes to catalyze four consecutive reactions: condensation of a fatty acyl-CoA with malonyl-CoA, reduction of the resulting 3-ketoacyl-CoA, dehydration of the 3-hydroxyacyl-CoA to 2,3-enoylacyl-CoA followed by a final reduction step. The first and rate limiting step [7] is catalyzed by elongase of very long chain fatty acids (Elovl). The six known mammalian elongases exhibit differential tissue expression [8]. In addition to Elovl 1-6, DNA sequence in Genbank (Genbank accesion number BC005602) suggests that there is a seventh member of the Elovl family which has similarity to Elovl1.

Three of the characterized enzymes elongate saturated fatty acids: Elovl1 synthesizes fatty acids with 26 carbon atoms (C26), Elovl3 elongates C16-C22 substrates and Elovl6 extends C12-C16 fatty acids. Polyunsaturated fatty acids, C20-C22, and C18-C20, are elongated by Elovl2 and Elovl5, respectively. Despite the use of similar approaches to those which led to successful identification of the substrates for the five characterized elongases, the substrate/product specificity of Elovl4 still remains unknown [9].

A role for ELOVL4 in the synthesis of docosahexaenoic acid (DHA, 22:6n-3), which represents approximately 50% of the fatty acids in retinal outer segment phospholipids [10], has been proposed [1]. DHA is synthesized from dietary essential linolenic acid (18:3n-3) in a series of three elongation steps [11], but to date no evidence has been presented to define a role for ELOVL4 in this pathway. In addition to DHA, the retina also contains C24-C36 polyenoic fatty acids, present in photoreceptor outer segment phosphatidylcholines [12], which are synthesized from eicosapentaenoic acid (20:5n-3) [13]. Reduced retinal synthesis of very long chain fatty acids, such as DHA as well as C24-C36 polyenoic fatty acids, as a result of a mutated ELOVL4 could play a role in the pathogenesis of STGD3.

Both cell biology and animal model studies have been performed to try to understand the function of Elovl4. In vitro expression of an *ELOVL4* transgene with a five-bp STGD3 deletion showed translocation of the mutated protein out of the ER [14,15]. When co-expressed, the mutant protein bound wt Elovl4 protein and carried it from the ER [16-18]. These findings suggest that the STGD3 mutation has a dominant negative effect which might lead to reduction in the levels of the currently unidentified ELOVL4 lipid products.

Results from studies in three different animal models of STGD3 have been reported recently. Studies in transgenic mice expressing the mutant STGD3 form of ELOVL4 in photoreceptors showed lipofuscin accumulation, retinal degeneration and reduced ERG signals [19], all features of human STGD3. In a second study, to address the physiological role of Elovl4, *Elovl4-*knockout mice were generated [20]. Retinas of heterozygous knockout mice demonstrated essentially normal retinal ERG function and minimal morphological abnormalities. The retinas of these mice had, however, a reduced content of several mono-unsaturated C16-C24 fatty acids. Homozygous knockout pups could not be obtained in this study. The third model reported is a heterozygous knock-in that introduced the five-bp STGD3 deletion in the mouse *Elovl4* gene [21]. At an age of 7-10 months, these mice had increased ERG signals, shortened photoreceptor outer segments without any loss of cell bodies, reduced Elovl4 mRNA levels, and a reduced content of several C18-C24 mono-, penta- and hexa-unsaturated fatty acids. No information about homozygous knockin mice was reported for this last study.

We have also generated a genetic knockin mouse model of STGD3. However, we chose to generate gene-knockin mice that carry not only the human 5-bp STGD3 pathogenic deletion in exon 6 of the mouse *Elovl4* gene but also two downstream single nucleotide substitutions. This design resulted in generation of an *Stgd3* allele that coded for a truncated mouse Elovl4 protein which also contained an aberrant 8-amino acid C-terminus identical to that encoded by the human pathogenic *STGD3* allele [1]. Our heterozygous Stgd3 mice reproduced retinal features of the human disease, lipofuscin accumulation and reduced vision, thus validating these animals as a mouse model suitable for studying the biochemical basis of STGD3 pathogenesis. One hypothetical STGD3 pathogenic mechanism is a "loss of function" mechanism resulting in deficiency of selected lipids, the putative Elovl4 products, in Elovl4-expressing tissues. Support for this mechanism is provided by the results of our analysis of epidermal lipids in neonatal homozygous Stgd3 mice which revealed a complete absence of acylceramides, a unique group of compound lipids containing C30-C40 fatty acids.

## Methods

### Generation of *Stgd3*-gene knockin mice

**Figure 1:**
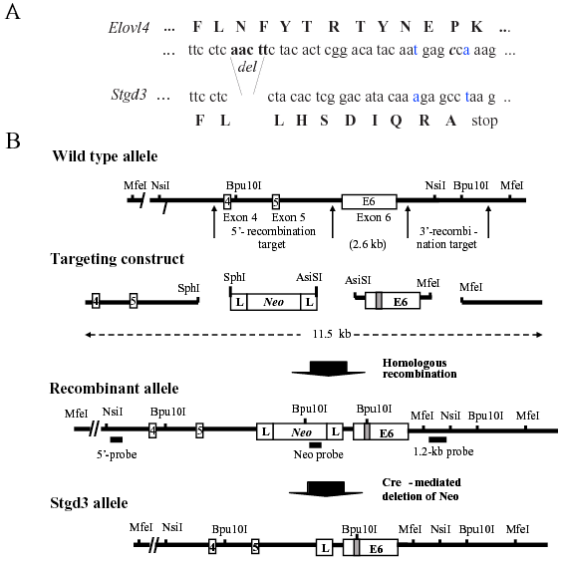
Gene-targeting strategy for generation of *Stgd3*-gene knockin mice. **A**: Partial nucleotide sequence from exon 6, and the encoded amino acids (capital letters), of the wt (*Elovl4*) and mutant (*Stgd3*) alleles. Shown are the mutations introduced into the *Elovl4* sequence prior to assembly of the targeting construct. Five nucleotide base pairs, corresponding to those absent in Stargardt-3 patients, were deleted (del). Two point mutations (the altered and substituted nucleotides are shown in blue) were introduced in the sequence downstream of the deletion to generate a new C-terminal sequence that is identical to that found in STGD3 patients. **B**: Schematic maps of a portion of the *Elovl4* allele encompassing exons 4, 5, and 6, the targeting construct, recombinant allele and the final *Stgd3* allele. Restriction sites used for cloning and for Southern blot verification of recombination and deletion events, as well as the location of the Southern probes are included. Mutations in exon 6 are shown schematically as a shaded box, within which the new *Bpu*10I restriction site introduced during sequence manipulation is shown. Homologous recombination of the 11.5-kb targeting construct generates a recombinant allele containing exon 6 with the Stgd3 mutation, a neomycin selection cassette (*Neo*) flanked by lox P sites (L), and 4.2 kb and 3.1kb of 5'- and 3'-targeting sequence, respectively. Breeding of recombinant Neo/wt mice with *Cre*-transgenic mice leads to Cre-mediated deletion of the *Neo* cassette and generation of the mice carrying the final *Stgd3* allele.

The targeting construct contained 4.2-kb of 5'-recombination target, a 1.6-kb loxP-flanked *Neo* cassette, 2.6-kb of genomic DNA containing the 2.1-kb *Elovl4* exon 6 sequence with the human STGD3 mutation, and 3.1-kb of 3'-recombination target (Figure 1B). Mouse DNA sequences were amplified using 128SvEv mouse DNA and the High Fidelity PCR System (Stratagene, La Jolla, CA) and cloned, together with the *Neo* cassette, into a PCR-XL-TOPO vector (Invitrogen, Carlsbad, CA). The unique restriction sites (*Sph*I, *Asi*SI and *Mfe*I, Figure 1B) used for cloning were incorporated in the sequence of PCR primers. Prior to targeting construct assembly, the PCR product with the *Elovl4* exon 6 sequence was mutated using internal PCR primers containing the human STGD3 mutation. The final 15-kb targeting construct was linearized using the vector-located *Sma*I site, and electroporated into 129SvEv embryonic stem cells, yielding 360 clones that survived G418 selection. Southern blot analysis of stem cell DNA digests detected three clones with homologous recombination. One of these, clone number 2F4, was injected into C57BL/6 blastocysts, resulting in the birth of 33 high-percentage male chimeras by coat color. Eight chimeric males were bred with 129SvEv females. PCR genotyping of their F1 offspring revealed that six males transferred the recombined allele with the *Neo* cassette to the next generation of Neo mice. To remove the loxP-flanked *Neo* cassette and obtain Stgd3 mice, the progeny of male number 3 were bred with *Cre*-transgenic mice, strain 129S1-Hprt^TM^ (Cre)Mnn/J (Jackson Laboratory, Bar Harbor, ME).

Electroporation, culture of embryonic stem cells and generation of chimeric mice were all done by The UT Southwestern Transgenic Technology Center. Mice were housed in The UT Southwestern Animal Resource Center under a 12 h light/12 h dark cycle and fed ad libidum a standard chow diet. All experiments were approved by the Institutional Animal Care and Use Committee of The UT Southwestern Medical Center, Dallas, TX.

### Southern blot analysis of knockin mice

Tail DNA samples were digested with *Nsi*I or *Bpu*10I (New England Biolabs, Beverly, MA), separated on an agarose gel, transferred to Hybond N^+^ membrane (GE Healthcare, Piscataway, NJ), and subjected to Southern blot analysis using P32-labeled DNA probes: a 453-bp 5'-external probe, a 284-bp Neo probe, or a 1.2-kb 3'-internal probe (Figure 1B).

### Polymerase chain reaction genotyping of mice

After founder mice were characterized by Southern blot analysis, further genotyping of their progenies was performed by PCR using tail DNA. The wt and Stgd3-mutant sequences (with or without the *Neo* cassette) were detected by PCR reactions run for 30 cycles of 15 s at 94 °C, 15 s at 60 °C, and 30 s at 72 °C using the sense primer 5'-GCA ATC ACT AGA ATG CCC TTG CTG AGC AGG TG-3' for both reactions, and antisense primer 5'-GGC TCA TTG TAT GTC CGA GTG TAG AAG TTG-3' for wt sequence and 5'-GCT CTT TGT ATG TCC GAG TGT AGG AGG A-3' for mutant sequence. Products of 332 and 327 bp were obtained for the wt and mutant sequences, respectively. The presence of a *Stgd3*-allele with a *Neo* deletion was detected as an 84-bp PCR product following 35 cycles of 15 s at 94 °C, 15 s at 65 °C, and 15 s at 72 °C using sense primer 5'-CGC GCC ATC GAT GGA TCC GGT ACC ATA ACT TCG-3' and antisense primer 5'-TGG TTT CCC CAA GGC AGT GCG ATC GCA TAA CTT CG-3'. The *Cre* transgene was detected by the presence of a 102-bp PCR product following 35 cycles of 30 s at 94 °C, 15 s at 56 °C, and 15 s at 72 °C, using primers designed by The Jackson Laboratory: sense 5'-GTG AAA CAG CAT TGC TGT CAC TT-3' and antisense 5'-GCG GTC TGG CAG TAA AAA CTA TC-3'.

### Nuclease protection assay

Total RNA was extracted from individual samples of mouse tissues using RNA-Stat60 reagent (Tel-Test, Inc., Friendswood, TX) according to the manufacturer's protocol. RNA samples were hybridized to an antisense [α-^32^P]UMP-labeled 322-nucleotide (N) RNA probe containing 182-N sequence of mouse Elovl4 mRNA (nucleotides number 840-659). After hybridization and S1 nuclease digestion, protected fragments were analyzed by electrophoresis on 8% polyacrylamide gels containing 8 M urea, as described previously [22]. The 182-N riboprobe fragment protected by Elovl4 (wt) mRNA, and two riboprobe fragments (containing 126 Ns and 51 Ns) protected by Stgd3 (mutant) mRNA were quantified on a Typhoon 9410 phosphoimager (Amersham Biosciences Corporation, Piscataway, NJ) and normalized for their content of radioactive UMP nucleotide.

### Analysis of A2E and its precursors

Mice were euthanized using halothane in the middle of the light phase of the day/night cycle. Eyecups were collected, extracted with chloroform and isolated retinal fluorophores analyzed as previously described [23].

### Electroretinogram analysis

Mice were dark adapted overnight with eyes dilated by topical application of 0.25% Isopto Hyoscine. Next day, after repeated eye dilation, mice were anesthetized by intraperitoneal injection of a saline solution containing Ketamine (200 mg/kg body weight) and Xylazine (10 mg/kg body weight). A Burian-Allen lens placed on one cornea was referenced to a needle electrode in the scalp. A needle electrode in the tail served as ground. To stabilize body temperature during testing, mice were placed between two heating pads. Rod and cone signals were recorded following ISCEV standard protocol. Signals were amplified (Tektronix AM502 differential amplifier; x10,000; 3 dB down at 2 and 10,000 Hz), digitized (sampling rate=1.25 to 5 kHz) and averaged on a personal computer. Two different flash stimulators were utilized. A Grass photostimulator provided short-wavelength 10 msec flashes (Wratten 47A: max=470 nm, half-bandwidth=55 nm) from-3.88-1.27 log scot td-s. A Novatron flash unit produced high-intensity 1.3 msec flashes from 0.9-3.65 log scot td-s. A steady background (3.2 log ph td) was used to isolate cone responses.

### Histology of mouse eyecups and skin

Mice were euthanized using halothane. Eyecups were collected from adult mice, and the head and skin from the dorsal trunk of new-born mice. Tissue samples were fixed overnight in phosphate buffered saline (PBS), pH 7.4, containing 4% formaldehyde. Then, they were dehydrated, paraffin embedded, and sectioned prior to staining with hematoxylin and eosin, using established procedures [24].

### Skin permeability assay

New-born mice were euthanized using halothane. Tails were collected for PCR genotyping and bodies were tested for skin barrier function according to [25] with one modification. Instead of a methanol wash, bodies were rinsed in PBS. Then, they were immersed in a PBS buffer containing 0.1% toluidine blue for 2-4 h at room temperature, washed briefly with PBS and photographed.

### HPLC/mass spectrometric analysis of epidermal lipids

Excised dorsal skin of new-born mice was immersed in PBS containing 10 mM EDTA at 37 °C for 45 min to separate the epidermis from the dermis as described previously [26]. Epidermal samples were snap frozen in liquid nitrogen and stored at -80 °C until analyzed. Samples were homogenized in 1.5 ml of a chloroform/methanol mixture (1:2 v/v) using a glass homogenizer, extracted overnight at 37 °C and centrifuged in a glass tube. The pellet was re-extracted overnight with 1.5 ml of a chloroform/methanol mixture (2:1 v/v) and centrifuged as before. The combined extracts were evaporated under argon and dissolved in chloroform/methanol (1:1 v/v) at a concentration of 0.1-0.5 mg/mL and fractionated by high performance liquid chromatography (HPLC) as described by [27]. Mass spectrometric (MS) analysis was performed using a LCQ Deca XP Max MS^n^ spectrometer (Thermo Electron Corporation, San Jose, CA) equipped with an atmospheric pressure chemical ionization ion source. Full MS spectra between m/z values of 100 and 2000 were collected in both the negative and positive ion modes. For positive ion mode analyses, the following conditions were applied: source voltage = 3.75 kV, source current=5 A, vaporizer temperature=375 °C, sheath gas (N_2_) flow=25 arbitrary units, auxiliary/sweep gas flow=4 arbitrary units, capillary voltage=12V, capillary temperature=300 °C. In negative mode experiments, the following conditions were used: source voltage=1.25 kV, source current=4.5 A, vaporizer temperature = 375 °C, sheath gas (N_2_) flow=25 arbitrary units, auxiliary/sweep gas flow=4 arbitrary units, capillary voltage = -15V, capillary temperature=300 °C.

Structural analysis of ceramides was performed using direct infusion of samples at a flow rate of 5-10 μl/min. Parent ions were subjected to collision induced dissociation by helium at 33-45% relative energy and resulting spectra accumulated for 1-2 min. MS experimental parameters were as described before.

## Results

### Generation of *Stgd3*-gene knockin mice

An *Elovl4*-targeting construct was generated in which exon 6 sequence was mutated to introduce a human disease-associated deletion, with an exact replication of the new C-terminal peptide sequence of the truncated human protein (Figure 1A). The human pathogenic 5-bp deletion in *ELOVL4* causes a frame shift which both removes an ER retention signal and truncates the 49-amino acid C-terminus to an aberrant 8-amino acid C-terminus (LHSDIQRA). To achieve this same truncated aberrant C-terminus in *Stgd3*-gene knockin mice, two point substitutions were introduced in the nucleotide sequence downstream of the deletion site (Figure 1A). Without these additional point mutations the truncated C-terminus would be shorter by only six amino acids (LHSDIQ). The final construct was then used to generate Stgd3 mice (Figure 1B).

**Figure 2:**
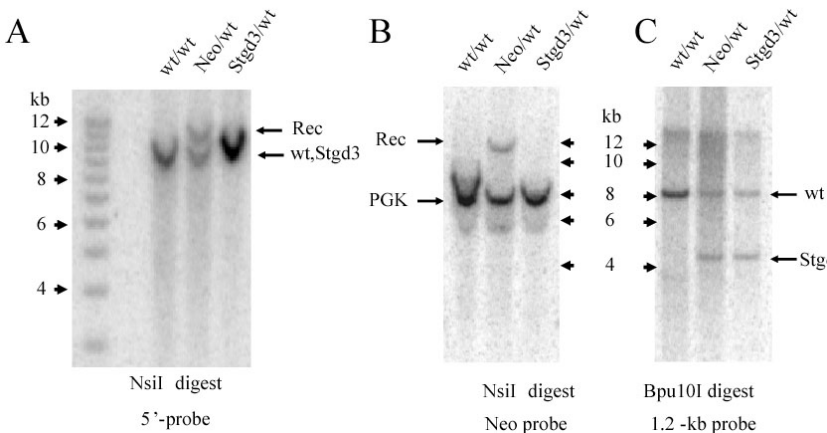
Southern blot verification of Stgd3 mouse genotype. **A**: Genomic DNA was digested with *Nsi*I and probed with a 5'-probe located upstream of the recombinant targeted site (see Figure 1). A signal from the *wt* allele as well as an extra band (Rec), derived from the recombinant allele, were detected in the Neo/wt DNA digests. The size of the Rec band was consistent with the 11.8-kb size predicted for the homologous recombination event, confirming the presence of recombination in these mice. In the Stgd3/wt DNA digests, the size of the recombined band was reduced due to loss of the *Neo* cassette with the result that the *wt* and *Stgd3* allele-derived signals were not resolved. **B**: Hybridization with the Neo probe detected the presence of the Neo signal in the Neo/wt but not in Stgd3/wt DNA digests, confirming recombination of the targeting sequence and Cre-mediated excision of the *Neo* cassette, respectively. The Neo probe contained a mouse phosphoglycerate kinase (*PGK*) promoter and thus also detected the endogenous *PGK*-gene sequence in all mouse DNA samples. **C**: Introduction of the STGD3 mutation into *Elovl4* exon 6 generated a new *Bpu*10I restriction site in this exon. The presence of the STGD3-mutation in the DNA of Neo/wt and Stgd3/wt mice was confirmed by the detection of a new 4.3-kb *Bpu*10I restriction fragment that was absent from wt/wt DNA.

Southern blot analysis of genomic DNA confirmed homologous recombination of the targeting construct in knockin mice, the presence of the *Neo*-cassette used for ES cell clone selection in Neo mice and its absence in the final Stgd3 mice (Figure 2). The mutations engineered into the *Elovl4* gene introduced a *Bpu*I restriction site into exon 6. The resulting detection of a new hybridization band following Southern blot analysis of *Bpu*I-digested tail DNA further confirmed the presence of the *Stgd3* allele in Stgd3 mice (Figure 2C). The *Stgd3* allele codes for a shortened Elovl4 protein that contains a wt N-terminal sequence of 263 amino acids and a C-terminus downstream of the mutation containing only eight amino acids. In summary, analysis of genomic DNA validated our Stgd3 mice as a genetic animal model that carries the human pathogenic STGD3 mutation in the mouse *Elovl4* gene.

### Heterozygous Stgd3 mice reproduce retinal features of human STGD3

**Figure 3:**
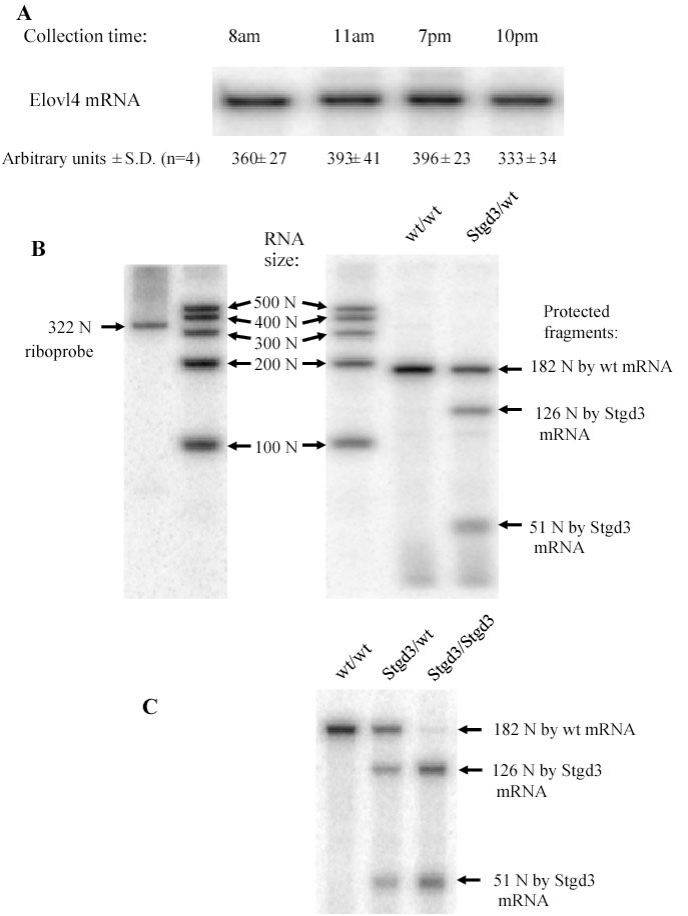
Expression of Elovl4 and Stgd3 mRNAs in mouse eyecups. S1-nuclease protection assay, using a 322-nucleotide (N) riboprobe, detected a 182-N fragment protected by Elovl4 (wt) mRNA and two fragments (126 Ns and 51 Ns) protected by Stgd3 (mutant) mRNA. **A**: Elovl4 mRNA levels in wt/wt retinas remained constant through the day/night cycle. **B**: Stgd3/wt retinas from 1-month-old mice expressed equivalent amounts of both wt and Stgd3 mRNAs (ratio 1.02±0.06; mean±SD, n=5). The level of Elovl4 mRNA in these mice was a half (51%±6; mean±SD, n=4) of that detected in the wt/wt littermates. RNA size standards in a range of 100 N-500 N are shown. **C**: The epidermal level of mutated Elovl4 mRNA (Stgd3 mRNA) in neonatal Stgd3/Stgd3 mice is comparable to the Elovl4 mRNA levels in wt/wt and Stgd3/wt littermates.

The heterozygous Stgd3 animals displayed no obvious gross morphological or behavioral alterations when compared to their wt littermates. Since STGD3 is a dominant retinal disease, we examined the eyes of our generated mutant mice for alterations. First, the mRNA levels derived from both the *wt* and *Stgd3* alleles were measured by an S1-nuclease protection assay that discriminated between and allowed quantification of both wt and mutant Elovl4 mRNAs. In contrast to some retinal mRNAs, Elovl4 mRNA levels did not undergo diurnal changes in mouse retinas (Figure 3A). Also, the level of wt Elovl4 mRNA in heterozygous Stgd3 retinas was not affected by the *Stgd3* mutant allele, since it was found to be half of the Elovl4 mRNA level that was present in wt retinas (Figure 3B). This shows a lack of any regulatory mechanisms that induce over-expression from the wt *Elovl4* allele to compensate for the presence of the mutated allele. The Stgd3 mutation also had no effect on stability of mutated Elovl4 mRNA since the retinas of heterozygous Stgd3 mice expressed equal amounts of both mutated and wt Elovl4 mRNAs. All these data suggest that the STGD3-mediated pathophysiology involves steps that are downstream of Elovl4 mRNA expression.

STGD3, similar to Stargardt disease-1 and age-related macular degeneration, is characterized by accumulation of lipofuscin in the retinal pigmented epithelium [5,6]. Its main fluorescent component is N-retinylidene-N-retinyletanolamine (A2E), a cytotoxic product of condensation of phosphatidylethanolamine with all-trans-retinaldehyde [28,29]. Also present are the A2E precursors: dihydro-N-retinylidene-N-retinylphosphatidylethanolamine (A2PE-H_2_), and N-retinylidene-N-retinylphosphatidylethanolamine (A2PE) [23]. In vivo, these precursors are slowly converted into A2E, resulting in A2E accumulation in retinal pigmented epithelium [23]. All these compounds were found in the eyecups of 9-month-old heterozygous Stgd3 mice. While the levels of A2E were 13% higher in mutant eyecups compared to that in the wt littermates, this difference was not sufficient to reach statistical significance (Table 1). In contrast, compared to the wt littermates, the amounts of both A2E precursors were significantly higher in the heterozygous Stgd3 mice, 87% more for A2PE-H_2_ and 31% more for A2PE. These analyses are consistent with the conclusion that our heterozygous Stgd3 mice replicate the lipofuscin accumulation which is one of the early hallmark features of the human STGD3 pathology [5,6].

**Table 1:** Levels of A2E and its precursors in eyecups of 9-month-old wt/wt and Stgd3/wt mice.

	wt/wt	Stgd3/wt	significance
A2E	7.1±1.0	8.0±0.7	p<0.125
A2PE-H2	3.0±1.1	5.6±1.0	p<0.003
A2PE	5.4±1.3	7.1±0.9	p<0.04

Chloroform extracts from eyecups of individual mice were analyzed for retinal fluorophores using high performance liquid chromatography as described previously in reference [23]. Levels of A2E and its precursors (A2E-H2 and A2PE) are expressed as means in pmoles per eyecup±standard deviation. Six mice were analyzed per group.

**Figure 4:**
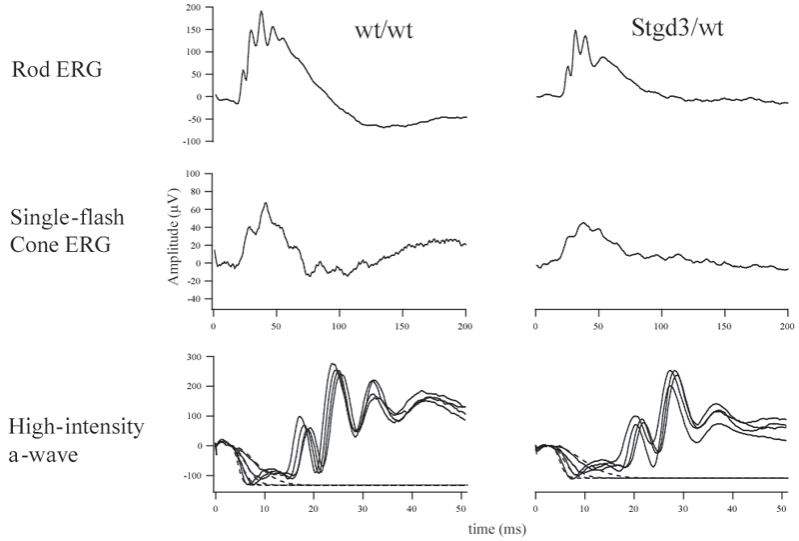
Electroretinographic analysis of heterozygous Stgd3 mice. Representative rod and cone electroretinograms, and a-wave responses are presented for 8-month-old Stgd3/wt mice and their wt/wt littermates. Electroretinographic parameters obtained from all mice (n=6 per group) examined for the study are summarized in Table 2.

Another feature of STGD3 is the juvenile-onset of visual loss which in older patients may be accompanied by reduced ERG signals [5,6]. Legal blindness occurs around a mean age of 20 years but patients start to lose vision in their teenage years [30]. We examined visual functions in our mutant mice using ERG analysis. When compared to their wt littermates, the 8-month-old heterozygous Stgd3 mice showed a reduction of their ERG responses to light, measured by the maximum rod b-wave amplitude and the maximum rod a-wave amplitude expressed as the maximal amplitude of the rod component (Figure 4, Table 2). The changes in the mutant cone-derived component of the ERG, though trending lower, were not statistically significant compared to that recorded in the wt mice (Figure 4, Table 2). In summary, the heterozygous Stgd3 mice reproduced two retinal features of the human disease: lipofuscin accumulation and reduced vision.

**Table 2:** Electroretinographic analysis of 8-month-old wt/wt and Stgd3/wt mice.

Variable	wt/wt	Stgd3/wt	Significance
Rod amplitude (μV)	164±33	100±19	p<0.002
Cone amplitude (μV)	63±22	44±10	p<0.09
Rmp3 (μV)	134±21	85±20	p<0.003
S	98±26	105±28	p<0.62

Electroretinographic parameters were derived from analysis of rod and cone electroretinograms (representative electroretinograms see Figure 4). Shown in this table are the values for rod amplitude obtained using single blue flash (-0.5 log scot td/s) and for cone amplitude obtained using single white flash on a rod-saturating background. To calculate the maximal rod response (Rmp3) and the amplification constant (S), the leading edge of the a-waves was fit as an ensemble [53] using the Lamb and Pugh model [54] for the activation phase of the phototransduction cascade. Values are means±standard deviation. Six mice were analyzed per group.

**Figure 5:**
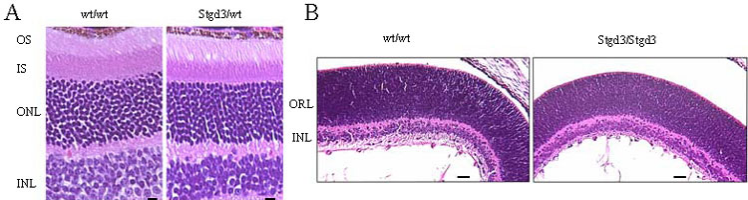
Light micrographs of hematoxylin and eosin stained sections of Stgd3 mouse retinas. **A**: Retinal sections from 8-month-old heterozygous Stgd3 mice and their wt littermates show similar thickness of photoreceptor outer segments (OS), inner segment (IS), outer nuclear (ONL) and inner nuclear (INL) layers. No significant changes in numbers and morphology of photoreceptors are evident. Scale bar represents 10 μm. **B**: Retinal sections from neonatal homozygous Stgd3 mice and their wt littermates show similar retinal histology, with distinct INL and larger outer retinal layers (ORL). The ORL contains the photoreceptor cell bodies which at this stage of development have not elaborated outer segments. Scale bar represents 100 μm.

These changes were not, however, accompanied by retinal degeneration. A light microscopy examination of retinal sections from 8-month-old heterozygous Stgd3 mice revealed no alterations in morphology when compared to the retinas of wt littermates (Figure 5A). All layers of the retina were present and no significant reduction in either the length of photoreceptor outer segments or the number of photoreceptor cell bodies was noted at this age. Additional support for the unchanged numbers of photoreceptors in 1-year-old Stgd3 mice came from quantitative analysis of Elovl4 mRNA levels. Total (combined wt and Stgd3) Elovl4 mRNA levels in heterozygous Stgd3 retinas were the same as the wt Elovl4 mRNA levels in wt retinas (ratio: 1.08±0.07, mean±SD, n=5).

### Homozygous Stgd3 mice die after birth with symptoms of a skin barrier defect

Analysis of phenotypic changes in homozygous Stgd3 mice was limited because of their neonatal lethality. Genotype analysis of 3-week-old progeny from initial breeding of heterozygous Stgd3 parents detected heterozygous but no homozygous offspring. However, when progeny were genotyped immediately following birth, pups with the predicted Mendelian 1:2:1 genotype distribution were identified. Genotyping of newborn pups from 8 litters identified 15 wt/wt, 27 Stgd3/wt and 13 Stgd3/Stgd3 pups. Thus, homozygous Stgd3 pups were born at the expected frequency but died after birth. At birth, the homozygous Stgd3 neonates were indistinguishable from their littermates but shortly thereafter they became lethargic and had erythematous, shiny and shriveled skin. All homozygous Stgd3 pups died within 6-12 h after birth.

Photoreceptor defects could not be examined in homozygous Stgd3 mice because retinal development is incomplete in neonatal mice. While photoreceptor cell bodies are present in neonate retina, they have not elaborated outer segments. In homozygous Stgd3 retina, however, the outer retinal layer and inner nuclear layer are present, displaying a normal morphology comparable to that of wt neonatal retina (Figure 5B).

**Figure 6:**
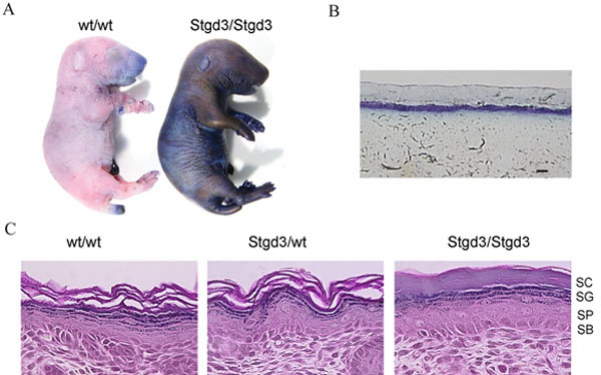
Defective skin barrier in neonatal homozygous Stgd3 mice. **A**: Skin permeability assay. Euthanized neonatal pups were immersed in a toluidine blue solution. The skin of homozygous Stgd3 mice, in contrast to their wt littermates, stained blue indicating a defect in skin barrier function. **B**: Light microscopy examination of a cross section of the dye-stained skin of the homozygous Stgd3 neonate shows penetration of the dye through the outer skin layers. **C**: Hematoxylin and eosin staining of homozygous Stgd3 skin shows the presence of all four epidermal layers: stratum corneum (SC), stratum granulosum (SG), stratum spinosum (SP) and stratum basale (SB). Note the more compact structure of stratum corneum in the mutant homozygous skin compared to the wt/wt and Stgd3/wt skin. Scale bar represents 100 μm in **B**.

The skin phenotype observed in homozygous Stgd3 mice is similar to that observed in knockout mice lacking keratinocyte transglutaminase [31] or acyl-CoA:diacylglycerol acyltransferase [26], both of which also die within a few hours of birth with symptoms of a skin barrier defect. Using a skin permeability assay [25], the homozygous Stgd3 mice also showed a defect in skin barrier function. When the bodies of euthanized neonatal mice were immersed in a toluidine blue solution, the dye stained the skin of homozygous Stgd3 animals but not wt (Figure 6A) or heterozygous Stgd3 (not shown) littermates. These findings suggest an underlying defect in the epidermis of the homozygous Stgd3 mice. Histological analysis of their skin showed that all four layers of the epidermis were present (Figure 6C). In addition, the amount of mutated Elovl4 mRNA present in homozygous Stgd3 epidermis was comparable to the amount of wt Elovl4 mRNA in wt epidermis (Figure 3C). This suggests no loss of *Elovl4*-expressing cells in homozygous Stgd3 epidermis. Morphological alterations were, however, evident in the outermost layer of the homozygous Stgd3 epidermis, which appeared compacted and lacked the laminations present in the wt and heterozygous Stgd3 epidermis. Alterations in the homozygous Stgd3 stratum corneum offer a plausible explanation as to why toluidine blue can penetrate and stain the inner epidermal layers (Figure 6B).

### Homozygous Stgd3 mice lack skin acylceramides

The multiple lipid lamellae that are located in the extracellular spaces between corneocytes in the stratum corneum are a vital component of the epidermal permeability barrier. These lipid enriched membranes, elaborated as part of the natural progression of differentiation of epidermal keratinocytes, are composed of cholesterol (25% by weight), free fatty acids (10-15%), and ceramides (45-50%) [32]. The epidermis is a very active site of lipid biosynthesis and previous studies have shown that inhibition of synthesis of the lipid components of the lamellae leads to perturbations in barrier function [33]. The presence of the disrupted skin barrier function in homozygous Stgd3 mice suggests that the lipid products of the Elovl4 enzymatic pathway may be important components of the lipid fraction of the stratum corneum. To pursue this, we isolated total unbound lipids from wt and homozygous Stgd3 neonatal epidermis and analyzed their composition using HPLC/MS.

**Figure 7:**
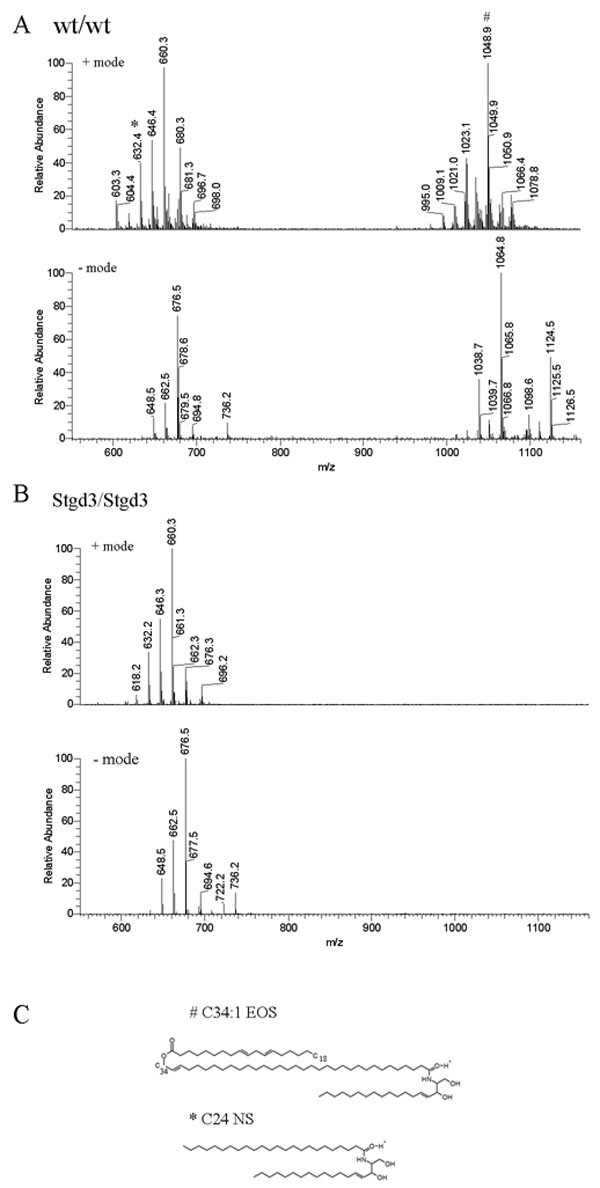
Epidermal ceramides in neonatal homozygous Stgd3 mice. Epidermal lipids were extracted and analyzed by high performance liquid chromatography/mass spectrometry (HPLC/MS). Full scan MS analysis of an HPLC ceramide fraction detected non-acylceramides (600-700 m/z) in both wt/wt (**A**) and Stgd3/Stgd3 epidermal extracts (**B**), but acylceramides (1000-1100 m/z) only in the wt/wt extract. Among non-acylceramides, we identified C24-NS ceramide based on a comparison with a commercial C24-NS standard (isotopic mass=649.63). M/z values for both were identical; 648.5 m/z in negative ion mode due to proton loss, and 632.4 m/z in positive ion mode due to proton gain and water loss. Also detected in both extracts were its CH_2_-homologs: C25-NS ceramide (662.5 and 646.4 m/z in negative and positive mode, respectively) and C26-NS ceramide (676.5 and 660.3 m/z, respectively). Among acylceramides in the wt/wt extract, we identified C34:1-EOS ceramide (isotopic mass 1066.00) based on an m/z value of 1064.8 in negative mode due to proton loss, and 1048.9 m/z in positive mode due to proton gain and water loss. Identity was further confirmed by fragmentation analysis (see Figure 9). Also present were CH_2_-homologs of C34-EOS containing varying degrees of fatty acid saturation (see Figure 8). **C** shows the structures of an acylceramide with a C34-fatty acid (C34:1-EOS) and a non-acylceramide with a C24 fatty acid (C-24-NS).

Both cholesterol and C16-C24 fatty acids, identified by m/z values of MS analysis and by comparison to commercially available standards, were detected in the lipid extracts of wt and mutant epidermis (data not shown). However, when the ceramide content of both extracts was compared, significant differences were observed (Figure 7A). Mammalian epidermis contains acylceramides and non-acylceramides [34-36]. Non-acylceramides consist of sphingosine or hydroxylated sphingosine linked to C18-C28 fatty acid. The acylceramides are built of sphingosine or hydroxylated sphingosine linked to an ω-hydroxy C30-C40 fatty acid which is esterified by linoleic acid.

MS analysis detected non-acylceramides in both wt and homozygous Stgd3 lipid extracts, but acylceramides only in the wt extracts (Figure 7). The molecular identities of non-acylceramides were deduced using their m/z values in negative and positive ion mode of MS analysis and by comparison to a commercially available C24-NS ceramide standard, containing a C24 non-hydroxy fatty acid which is amide-linked to sphingosine (see chemical structure in Figure 7C). In negative ion mode, the C24-NS standard (isotopic mass of 649.63) produced a peak at 648.5 m/z, which in positive mode was changed to 632.4 m/z as a consequence of water loss (-18) and gain of two protons (+2). Peaks of identical m/z values in both MS ion modes were observed for the wt and homozygous Stgd3 lipid extracts (Figure 7) providing evidence for the presence of C24-NS ceramide in both wt and mutant epidermis. Also detected in both extracts were its CH_2_-homologs as well as members of other classes of non-acylceramides that contain hydroxylated sphingosine and/or a varying chain-length fatty acid. Thus, based on MS analysis, all non-acylceramides identified in the wt epidermal non-bound lipids were also present in the homozygous Stgd3 epidermis.

**Figure 8:**
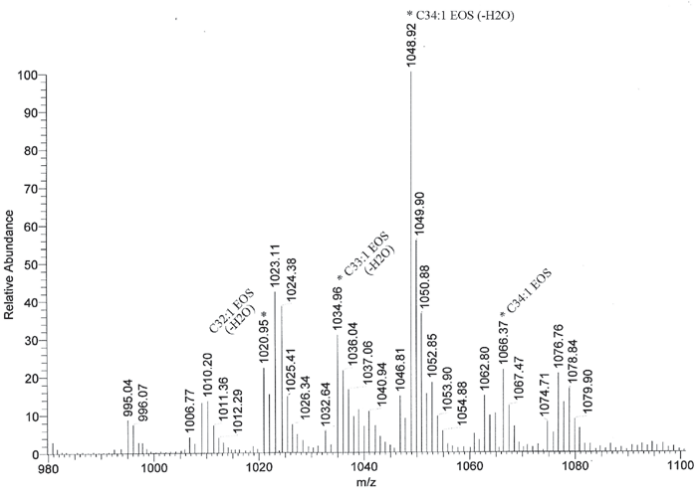
Acylceramides of wild-type mouse epidermis. Extended print-out of the acylceramides detected using positive ion mode MS analysis (Figure 7). It shows the 1066.37 m/z peak identified as C34:1-EOS by fragmentation analysis (see Figure 9) and the 1048.92 m/z peak of its dehydrated derivative. Signals 1020.95, and 1034.96 m/z correspond to C32:1-EOS, and C33:1-EOS, respectively. These are CH_2_-homologs of C34-EOS. Other peaks can be assigned as EOS ceramides containing varied degrees of fatty acid saturation or as minor acylceramide species (EOH and EOP) that contain an additional hydroxy group.

In contrast to the non-acylceramides, MS analysis revealed that all acylceramides were completely missing from the unbound lipid fraction of the homozygous Stgd3 epidermis. All acylceramides contain an unusually long chain C30-C40 fatty acid with an ω-hydroxy group esterified by linoleic acid [32,36]. No commercial standards were available to aid in identification of these species. Therefore, acylceramides were identified based on their m/z values, water loss by sphingosine ceramides in positive mode, and MS fragmentation analysis. The 1066.37 m/z peak of the negative mode MS was identified as C34:1-EOS ceramide (isotopic mass of 1066.00) which is composed of sphingosine linked to an ω-hydroxy monounsaturated C34 fatty acid esterified with linoleic acid (see chemical structure in Figure 7C). This ceramide loses water in positive mode MS to give a 1048.92 m/z signal (Figure 7A and Figure 8). This same C34:1-EOS ceramide, with identical MS signature, has previously been reported for mouse epidermis [37]. Our wt epidermal extracts also contain C32-C37 EOS ceramides which are CH_2_-homologs of C34:1-EOS (Figure 7A), as well as other acylceramides that contain fatty acids with varying degrees of saturation (Figure 8).

**Figure 9:**
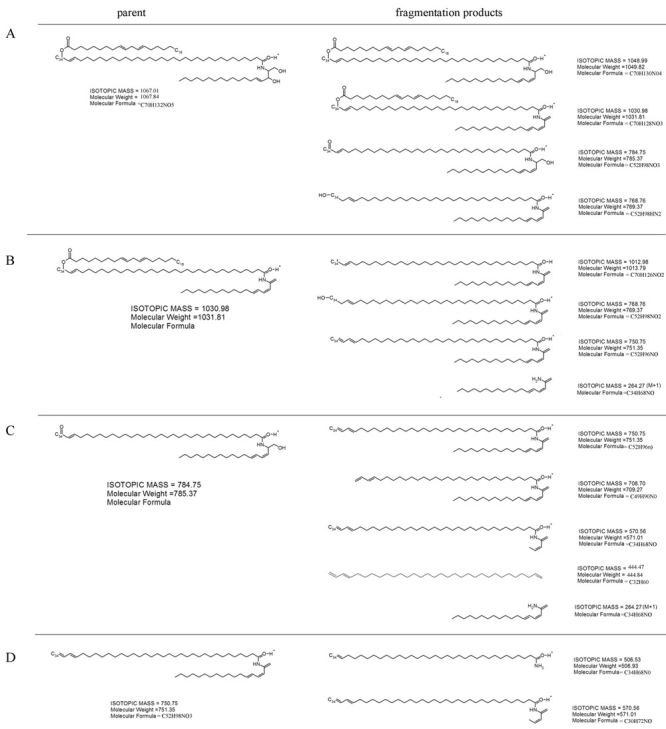
Fragmentation analysis of wild-type epidermal ceramide. The MS positive mode 1066.4 m/z peak (see Figure 7A) was fragmented by collision-induced dissociation and resulted in generation of 1048.1, 1030.6, 744.2, and 769.0 m/z daughter ions. Chemical structures for compounds of these molecular masses are provided in panel **A**: Further fragmentation of the 1030 m/z ion produced 1012.9, 768.6, 750.7, and 263.9 m/z signals (deduced compound formulas are shown in panel **B**). Transformation of the 768.6 m/z ion yielded 750.5 + 708.7 + 570.3 + 444.3 + 263.9 m/z ions (**C**), and fragmentation of the 750.5 m/z ion produced 571.1 and 506.2 m/z ions (**D**). The observed daughter ions are consistent with fragment compounds expected from C34:1-EOS ceramide which contains sphingosine linked to an ω-hydroxy C34 monounsaturated fatty acid that is esterified with linoleic acid.

Identification of the high molecular weight C34:1-EOS ceramide was further confirmed by performing MS analysis in positive ion mode on a successive series of fragmentation products derived from collision induced dissociation of the parent ion (Figure 9). Resulting daughter ions from fragmentation analysis were consistent with identification of the parent compound as a C34:1-EOS ceramide. The MS data, in summary, indicate that the epidermis in homozygous Stgd3 animals is completely lacking in acylceramides.

## Discussion

To study the physiological and pathophysiological functions of Elovl4, we have generated *Stgd3*-gene knockin mice. They are a genetic model of human STGD3 which is caused by mutations in the human *ELOVL4* gene. The *Elovl4* targeting construct was designed to not only introduce the human disease-associated 5-bp deletion but also to replicate exactly the human amino acid C-terminal sequence coded by the human pathogenic *ELOVL4* allele.

Heterozygous Stgd3 mice were found to have alterations that replicate both biochemical and functional features of the human pathology. The eyes of 9-month-old heterozygous Stgd3 mice, while not showing statistically significant changes in retinal A2E levels, did have increased levels of the A2E precursors, A2PE-H_2_ and A2PE. Recent studies have shown that these precursors are slowly converted in vivo into A2E in wt mice after intravitreal administration of A2PE-H_2_ [23]. Since A2E and its precursors are major fluorescent constituents of lipofuscin, this suggests that heterozygous Stgd3 mice replicate STGD3-associated retinal lipofuscin accumulation, and that they may represent a model of the early steps of this process. Previously, increased A2E levels have also been shown in transgenic mice over-expressing a human STGD3 protein [19] as well as in *abca4*-gene knockout mice [28,29], an animal model of the phenotypically similar Stargardt disease-1. A2E has been shown to be cytotoxic to retinal pigmented epithelial cells [38-40] and, when present, may play a role in the pathogenesis of macular degenerations, including age-related macular degeneration [41].

Heterozygous Stgd3 mice also showed defects in visual function, again replicating visual function alterations in STGD3 patients [5,6]. The decrease in rod b-wave amplitudes recorded in our mutant mice is similar to a decrease reported for transgenic mice that over-express human STGD3 protein in photoreceptors [19]. Although the heterozygous Stgd3 mice exhibit biochemical and functional changes in the retina, we do not see any significant morphological changes in the eyes of 8-month-old animals using light microscopy. Ongoing studies will determine whether morphological changes will be observed in much older animals. However, the lack of morphological change in our 8-month-old mutants agrees with the observation that heterozygous *Elovl4* gene-knockout mice had only minimal morphological abnormalities at the age of 17 to 19 months [20]. This lack of change, observed in both our Stgd3 mice and in the *Elovl4* gene-knockout mice, contrasts with the progressive, although limited, morphological changes reported in the retinas of another *Elovl4* 5 bp-deletion knock-in mouse model [21]. This difference between the two *Elovl4*-gene knockin mouse models is probably a result of several differences that exist between the two models.

Compared to the mice generated by Vassireddy et al. [21], our Stgd3 mice carry, besides the common 5-bp deletion, two additional point mutations to generate the STGD3 protein C-terminus. As a result the C-termini of both mutated Elovl4 proteins are different. Additionally, there are also other differences between these two mouse models. First of all, we inserted loxP in intron 5 of our Stgd3 mouse allele (Figure 1), with the result that the loxP sequence is not incorporated into the mutant Elovl4 mRNA. In contrast, the Vassireddy's mouse model was generated using a targeting vector which contained a loxP-flanked Neo-cassette inserted in exon 6. After Cre-mediated deletion of the Neo-cassette, this would result in the presence of one loxP sequence in exon 6 and, therefore, in the resulting mutant Elovl4 mRNA [21]. The presence of this additional 34-nucleotide sequence, consisting of two 13-bp inverted repeats [42], may explain the different levels of mutant Elovl4 mRNAs detected in both mouse groups. Vassireddy et al. [21] found a reduction by approximately a half of the levels of mutant Elovl4 mRNA compared to the wt Elovl4 mRNA in retinas of heterozygous mutant mice. In contrast, in our heterozygous Stgd3 mice the retinal levels of the mutant and wt Elovl4 mRNAs were the same (Figure 3B). Beside the above mentioned differences in nucleotide sequences and stability of mutant Elovl4 mRNAs, both mouse models are on different genetic backgrounds.

To investigate the biochemical effects of the mutant allele in our *Elovl4* 5 bp-deletion knock-in mouse model, we initially examined the effects on Elovl4 mRNA levels. Using a quantitative nuclease protection assay, we showed that expression of mRNA from the mutant *Stgd3* allele in heterozygous Stgd3 retinas does not lead to any up-regulation of expression from the wt *Elovl4* allele. In fact, the mRNAs derived from both, the *Stgd3* and *wt* alleles, were present at the same levels. There is, thus, no regulatory mechanism that detects and/or compensate at the Elovl4 mRNA level for the presence of the pathogenic *Stgd3* allele. Therefore, the pathogenesis involves a downstream pathway, most likely causing a deficit of Elovl4 protein and/or its products.

This conclusion is supported by the findings that all three different STGD3-associated mutations identified in the *ELOVL4* gene lead to protein truncation, with resulting loss of an ER retention signal [1]. Previous studies have shown that the human mutated ELOVL4 protein, when expressed in transfected cultured cells, bound to co-expressed human wt ELOVL4 protein and carried it from the ER [16-18], the site of fatty acid synthesis. The result of this dominant negative effect is a loss of the ER-located ELOVL4 protein. Additional possible outcomes of an abnormal ELOVL4 subcellular localization may be deficiency of ELOVL4 lipid products and/or a reduction in the wt and mutant ELOVL4 protein levels.

Further support for the dominant negative effect of the STGD3 mutation and resulting Elovl4 enzyme deficiency is suggested by the recently published studies in heterozygous *Elovl4* gene-knockout mice. Compared to their wt littermates, these mice had no remarkable changes in ERG [20]. This is in contrast to the reduced ERG signals in the heterozygous Stgd3 mice we report. One explanation for the ERG difference may be increased ER deficiency of Elovl4 protein in heterozygous Stgd3 mice as a result of its cellular miss-localization in the presence of the mutated Elovl4 protein [16-18].

Since the ER is the site of synthesis of very long chain fatty acids, an end-result of loss of ELOVL4 from there would be deficiency of the ELOVL4 enzymatic products, especially given that the elongation step is assumed to be the rate-limiting step in synthesis of long chain fatty acids [7]. That the fatty acid products of the *Elovl4* gene are important cellular constituents was suggested by the neonatal lethality observed in our homozygous Stgd3 mice and the recently reported inability to obtain homozygous *Elovl4*-gene knockout mice [20]. The most obvious change in the new-born homozygous Stgd3 mice was loss of skin barrier function. Whether a similar defect in skin function would be present in humans homozygous for the STGD3 mutation is unknown since we are not aware of any reported progeny from parents who are both heterozygous for the STGD3 mutation.

However, a similar skin phenotype has previously been described for mice with aberrant skin lipid metabolism caused by deletion of acyl-CoA:diacylglycerol acyltransferase-2 [26], fatty acid transport protein-4 [43], or ARNT-transcription factor [37], and for mice lacking keratinocyte transglutaminase which cross-links keratinocyte proteins producing insoluble membranous structures in the stratum corneum [31]. Mutations in keratinocyte transglutaminase have been found in some families with the autosomal recessive skin disorder termed lamellar ichtyosis [44-46]. Similar human skin pathologies may also involve *ELOVL4*. This conclusion is supported by observation that atopic dermatitis results from defective skin barrier function caused by significantly reduced levels of acylceramides [47] which are completely absent from the epidermis of our homozygous Stgd3 mice.

Epidermal keratinocytes synthesize acylceramide and non-acylceramide lipids [33]. Because homozygous Stgd3 mouse epidermis contains non-acylceramides, all four keratinocyte layers and high levels of mutated Elovl4 mRNA, we conclude that the *Elovl4-*expressing cells are still present in homozygous Stgd3 mouse epidermis and that the lack of acylceramides is a direct result of the STGD3 mutation in the Elovl4 protein. The compound acylceramide lipids, therefore, emerge as, previously unknown, products of the Elovl4 biosynthetic pathway. Deficit of these lipids in homozygous Stgd3 mice is most probably caused by a lack of one of their biosynthetic components. Homozygous Stgd3 epidermis was found to have a normal complement of non-acylceramide lipids, lipids which share ceramide backbones and in some cases linoleic acid in common with acylceramides. This suggests that it is absence of another acylceramide component that leads to their complete absence in homozygous Stgd3 mouse skin. Unique to acylceramides, as compared to non-acylceramides, and present in all classes of acylceramides, are very long chain fatty acids containing 30-40 carbon atoms. Our studies suggest that these fatty acids are the direct elongation products of Elovl4. Till now, no elongase has been shown to have a role in synthesis of such fatty acids. An inability to synthesize these long chain fatty acids would explain the complete absence of acylceramides from the lipid fraction of homozygous Stgd3 epidermis.

Acylceramide lipids are not found in the eye. However, other compound lipids containing very long chain C30-C40 fatty acids have been detected in the retina [12,13,48], brain [49] and testis [50], all tissues expressing high levels of Elovl4 mRNA [3,51]. In bovine retina such long chain fatty acids have been found in dipolyunsaturated phosphatidylcholines (PC) which also contain DHA at the sn-2 position [12]. These particular PC species have been shown to maintain a tight association with rhodopsin [52]. Ongoing studies will address whether these PC species are altered in STGD3 retinas.
